# Effectiveness of an anti-stigma training on improving attitudes and decreasing discrimination towards people with mental disorders among care assistant workers in Guangzhou, China

**DOI:** 10.1186/s13033-018-0259-2

**Published:** 2019-01-03

**Authors:** Jie Li, Yu Fan, Hua-Qing Zhong, Xiao-Ling Duan, Wen Chen, Sara Evans-Lacko, Graham Thornicroft

**Affiliations:** 1 The Affiliated Brain Hospital of Guangzhou Medical University (Guangzhou Huiai Hospital), NO. 36 Mingxin Road, Liwan District, Guangzhou, 510370 China; 2 Faculty of Medical Statistics and Epidemiology, School of Public Health, Sun Yat-sen Center for Migrant Health Policy, Sun Yat-sen University, Guangzhou, China; 3 Personal Social Services Research Unit, London School of Economics and Political Science, London, UK; 4 Centre for Global Mental Health, Institute of Psychiatry, Psychology and Neuroscience, King’s College London, London, SE5 8AF UK

**Keywords:** Treatment gap, Stigma and discrimination, Care assistant workers, Mental health, Low-and middle-income countries

## Abstract

**Background:**

Care assistant workers as a new pattern of care providers in China play an important role in bridging the mental health treatment gap. Stigma and discrimination against people with mental disorders among care assistant workers is a barrier which adversely influences mental health service delivery. However, programs aimed at reducing stigma among care assistant workers are rare in China.

**Methods:**

A total of 293 care assistant workers from four districts of Guangzhou, China were randomly divided into an intervention group (n = 139) and a control group (n = 154). The intervention group received anti-stigma training and the control group received traditional mental health training. Both trainings lasted for 3 h. Participants were measured before and after training using Perceived Devaluation and Discrimination Scale (PDD), Mental illness: Clinicians’ Attitudes (MICA) and Mental Health Knowledge Schedule (MAKS). Data were analyzed by descriptive statistics, *t*-test, Chi square test or Fisher’s exact test. Multilinear regression models were performed to calculate adjusted regression coefficient of the intervention on PPD, MAKS, and MICA.

**Results:**

There were significant lower scores on PDD and MICA in the intervention group after training when compared with the control group (both *P *< 0.001). No significant difference was found on MAKS total score between the two groups after training (*P *= 0.118). Both groups had better correct identification of schizophrenia, depression and bipolar disorder before and after training.

**Conclusions:**

These findings suggest that anti-stigma training may be effective in reducing the perception of devaluation-discrimination against people with mental illness and decreasing the level of negative stigma-related mental health attitudes among care assistant workers.

## Background

After decades of sustained global efforts, mental health is considered a global priority in the Sustainable Development Goals in 2015, which means mental health is now firmly included in the global development agenda [1, 2]. Mental disorders are closely correlated with high disability and premature mortality [3]. It is reported that mental illness accounts for 32.4% of years lived with disability and 13.0% of disability-adjusted life-years [4]. The treatment gaps mean that over 95% of people with common mental disorders in low- and middle-income countries (LMICs) do not receive effective treatment [5]. Human resource scarcity and ubiquitous stigma associated with mental disorders contribute to such disadvantage [6].

Mental disorders are different from some other diseases to some extent. For example, its assessment is mainly focused on professionals, not relying on advanced technology or medical equipment [7]. The shortage of mental health related specialist workers increases the difficulty in delivering mental health care in LMICs. Evidence-based practices recommend task sharing as an effective approach to overcome the treatment gap and to scale up mental health care [8–10]. The core of task sharing is to reallocate tasks from specialists to non-specialist workers. Kakuma et al. have given a report about human resources of mental health care, which describes three groups of mental health workforce, such as specialist workers (psychiatrists, psychologists, neurologists, et al.), non-specialist health workers (doctors, lay health workers, caregivers, et al.) and other professionals (teachers, community workers). Trained and skilled non-specialist health workers and other professionals could deliver effective mental health care and relieve human resource crises [11–13].

According to the current situation of China, more and more mental health service users receive mental health care in community, which motivate a new pattern of mental health care provider called the care assistant workers. Care assistant workers in China usually include primary health workers, community policemen, community cadres, volunteer and others. Primary health workers are usually general practitioners, public health doctors and nurses who provide general primary health care services. Community cadres are a group of relatively fixed people providing comprehensive services for the residents living in the community. These workers are established to assist mental health professionals to provide follow-up care, supervise medication compliance, support rehabilitation services, and to help patients and caregivers during crisis. Additionally, these workers are also supervised and monitored by the mental health professionals. The advantages of the care assistant workers are obvious. For example, the collaborations with different sectors could increase the detection of mental disorders and improve the mental health care delivery. What is more, sharing similar community culture with mentally ill persons, care assistant workers can handle certain health challenges and act as additional supporters for caregivers [14, 15].

Similar to other countries, severe mental disorders (SMD) in China are associated with long duration and high disability, which can increase family economical burden and the emotional costs of caregivers [16, 17]. Care assistant workers in China priority provide mental health care for people with SMD, which can provide a strong support for caregivers, a new gatekeeper for social harmony and a reasonable cost. This is also the product of the new social governance model based on collaboration, participation and common interests in current China. Patients with SMD, such as schizophrenia, schizoaffective disorder and bipolar disorder, who are assessed with a higher risk level, are equipped with a caregiver (always family members) and two care assistant workers, which is also called “1 + 2” model in China.

However, there is strong evidence suggesting that the ignorance of mental illness knowledge and prejudice and discrimination against people with mental illness among the care assistant workers are significant barriers in mental health care implementation [18–20]. These negative attitudes and discriminatory behaviors could lead to adverse consequences, such as unwillingness to provide mental health care, feeling impatient, spending less time on them, and paying unequal attention and substandard care for their physical health complaints and sometimes even ignore their human rights [21–24]. What’s worse, training programs for care assistant workers that integrate health education and stigma and discrimination reduction techniques are rarely implemented in China. Interventions are urgently needed to address the negative attitudes and discrimination among care assistant workers. Guangzhou, one of the megacities in China, has its own mental health service model, which named “PTSA” (Policy, Training, Service, and Assessment) [25]. Mental health training and assessment are important ingredients of this model. This study aims to evaluate an anti-stigma training program among these care assistant workers. We hypothesized that the anti-stigma intervention would reduce care assistant workers’ stigmatizing attitudes and increase their mental health knowledge after the training.

## Methods

### Risk assessment of patients with SMD in China

In order to strengthen the management and treatment of patients with SMD, the national community-based model named “686 Program” formulated the risk assessment questionnaire for patients with SMD, which could be used to assess patients’ risk assessment level [26].

There were 6 levels of the risk assessment questionnaire, which ranged from 0 to 5. Level 0 represents patients with no risks; Level 1 represents patients who presented with verbal threats and shouting, but no smashing behaviors; Level 2 represents patients who committed vandalism against property, which happened only at home and could be persuaded to stop; Level 3 represents patients with obvious vandalism against property which could have happened anywhere and could not be persuaded to stop; Level 4 represents patients who had persistent vandalism against property or person, including self-injury, suicide, and could not be persuaded to stop; Level 5 represents patients who committed violence against a person, including arson or explosion, whether at home or in public.

There were about 50,000 patients with SMD registered in the registry system of Guangzhou severe mental disorders management database. About one thousand patients (2%) were assessed as having a risk higher than level 3. Due to the limited resources, care assistant workers prioritized provision of mental health care to those with a risk score higher than 3, and had potential violence to themselves, family and society to some degree.

### Study design and participants

The study was conducted in Guangzhou Huiai Hospital on November, 2017. Ethics approval was obtained from the Research Ethics Committee of Guangzhou Huiai Hospital (Number 025, 2017). According to our previous study [27], two urban districts and two suburban districts were randomly selected based on their geographical locations in Guangzhou City. The four districts were then randomly divided into two groups, an intervention group and a control group. Each group consisted of an urban district and a suburban district.

All care assistant workers who worked in the selected districts and provided mental health services to SMD patients with risk assessment over level 3 were informed to take part in the voluntary training. Participants were asked to complete two standardized scales and a schedule before and after the training and they could leave the training at any time, but only those who finished the assessment before and after training could be included in the data analysis. Written informed consent was obtained from the participants after the procedure had been fully explained. A total of 293 participants (139 in intervention group and 154 in control group) were analyzed during this study.

### Interventions

Psycho-education was the main form for the study to provide the intervention. Both trainings in the two groups included three modules and the first two modules were the same. The first two modules included financial assistance policy of care assistant workers and mental health knowledge. The two same modules in intervention group were given by a social worker and a senior psychiatrist, and in control group they were given by a public health doctor and another senior psychiatrist. PowerPoint slides of the two modules between the two groups were the same. Trainers were trained by the research conductor (the first author) who was experienced in illustrating the PowerPoint slides to ensure the reliability and trainers were also supervised by the research conductor during the whole study and the two modules lasted for 2 h.

The third module in the intervention group was given the anti-stigma training, including stigma related to mental illness. Another senior psychiatrist gave training to the intervention group. The control group was given the traditional mental health training, including the insurance policies related with mental illness. A psychotherapist gave training to the control group. The third module lasted for an hour. Trainers were also trained by the research conductor and were also supervised by the research conductor during the whole study. Both two groups were trained with 3 h.

**Fig. 1:**
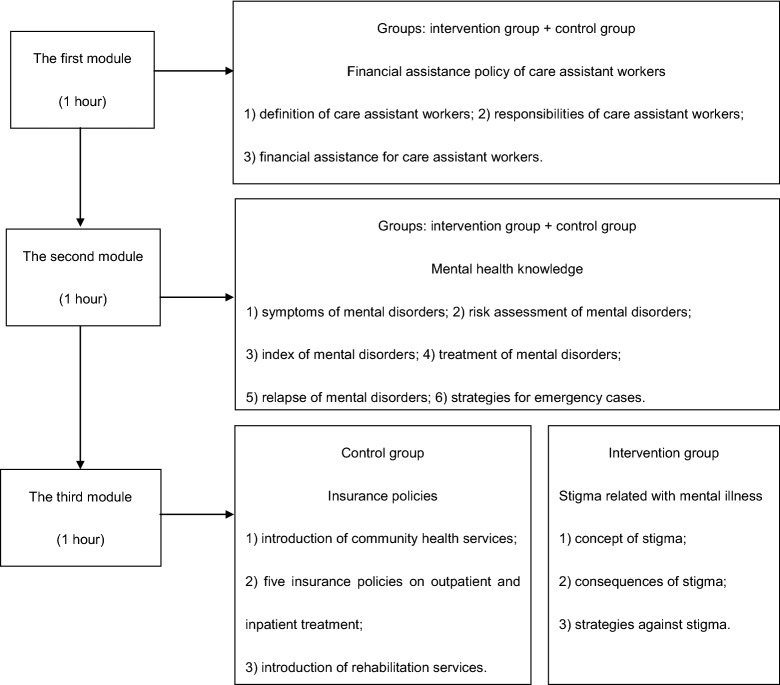
The modules of the training program

The main contents of the three modules are described below (Fig. 1):

Financial assistance policy of care assistant workers comprised three parts: (1) definition of care assistant workers; (2) responsibilities of care assistant workers; (3) financial assistance for care assistant workers. Mental health knowledge was comprised with six parts: (1) symptoms of mental disorders; (2) risk assessment of mental disorders; (3) index of mental disorders; (4) treatment of mental disorders; (5) relapse of mental disorders; (6) strategies for emergency cases. Insurance policies comprised three parts: (1) introduction of community health services; (2) five insurance policies on outpatient and inpatient treatment; (3) introduction of rehabilitation services. Stigma related with mental illness comprised three parts: (1) concept of stigma; (2) consequences of stigma; (3) strategies against stigma. During the anti-stigma training, the trainer listed some successful examples about the rehabilitation in patients with SMD and also delivered many advanced knowledge and experience, which could be meaningful in improving participants’ knowledge, attitude and behaviors. The lecture of the stigma related with mental illness was formulated mainly according to the WHO Mental Health Gap Action Program Intervention Guide [28]. Other lectures were formulated according to the national government recommended documents.

## Instruments

### Perceived Devaluation and Discrimination Scale (PDD)

There were 12 items in the Perceived Devaluation and Discrimination Scale (PDD), which was developed by Link. Each item is rated on a six-point scale which ranged from strongly agree to strongly disagree [29]. PDD was used to measure the extent to which a person believes that other people will devalue or discriminate against someone with a mental illness. A higher score indicated a stronger perception of devaluation-discrimination. During this study, we used the Chinese version of PDD, which has been tested with good validity and reliability (Cronbach α = 0.70) [30, 31].

### Mental illness: Clinicians’ Attitudes (MICA)

Mental illness: Clinicians’ Attitudes (MICA) was used to assess the participants’ attitudes towards mental illness and psychiatry [32]. The scale consisted of 16 items and a 6-point scale which ranged from strongly agree to strongly disagree. A higher score indicated a higher level of negative stigma-related mental health attitudes. During this study, we used the Chinese version of MICA, which has been tested with good validity and reliability (Cronbach α = 0.72) [26, 33].

### Mental Health Knowledge Schedule (MAKS)

Mental Health Knowledge Schedule (MAKS) consisted of 12 items and each item was rated on a 5-point Likert scale which ranged from strongly disagree to strongly agree [34]. The MAKS comprises two sections: the first 6 items are used to assess stigma-related knowledge where a higher total score indicates greater stigma-related mental health knowledge. The other six items (items 7–12) were used to assess participants’ correct identification of Depression, Stress, Schizophrenia, Bipolar Disorder, Drug Addiction and Grief as mental illnesses and to contextualize responses in part one. MAKS was a brief instrument which should be used in conjunction with other scales related with attitude and behaviour. In this study, MAKS was combined using with PDD and MICA.

### Statistical analysis

Statistical analyses were performed using IBM SPSS Statistics 20.0 (IBM Corporation, USA). To analyze the effectiveness of the intervention, firstly, pair-wise *t* tests were used to compare the differences in outcomes between intervention and control groups at baseline. Second, multilinear regression models were performed to calculate adjusted regression coefficient (*Ab*) of intervention on PPD, MAKS, and MICA with corresponding 95% confidence intervals (*CI*), while statistically significant demographics (education level and care assistant workers) and baseline scores (PDD and MICA) of outcome measures were adjusted. In the multilinear regression models, dummy variables were assigned to the confounding variable “care assistant workers”, with the “others” category was taken as the reference group. Significance was set at *P *< 0.05.

## Results

### Participant baseline characteristics

Before the study beginning, a total of 384 participants were invited (187 in intervention group and 197 in control group). As shown in Table 1, there were a total of 293 care assistant workers (139 in intervention group and 154 in control group) finished the assessment before and after the training. The response rate was 76.3% (74.3% in intervention group and 78.2% in control group). Care assistant workers consisted of primary health workers, community policemen, community cadres, volunteers and others, all of whom were responsible for different kinds of mental disorders, such as schizophrenia, schizoaffective disorder, paranoid mental disorders, bipolar disorder, mental disorders due to epilepsy, mental retardation with mental symptoms and other mental disorders. Before training, no significant differences were found between the two groups in age, sex, religion, race, disease management, and care willingness, except education level and care assistant workers (*P *< 0.05).

**Table 1:** Social-demographic characteristics (baseline)

Characteristics	Intervention group (n = 139)	Control group (n = 154)	*t*/*x*^2^	*P*
Age, years: mean (SD)	37.99 (9.07)	39.97 (10.29)	− 1.75	0.08
Race (Han), n (%)	137 (98.6)	151 (98.1)	0.11	0.74
Sex n (%)			3.42	0.08
Male	69 (49.6)	93 (60.4)		
Female	70 (50.4)	61 (39.6)		
Education level, n (%)			6.68	0.01
High school or below	15 (10.8)	34 (22.1)		
College degree or above	124 (89.2)	120 (77.9)		
Religion, n (%)			0.04	0.98
No	132 (95.0)	147 (95.5)		
Christianism	2 (1.4)	2 (1.3)		
Buddhism	5 (3.6)	5 (3.2)		
Disease management, n (%)			6.11	0.42
Schizophrenia	102 (73.4)	107 (69.5)		
Schizoaffective disorder	3 (2.2)	5 (3.2)		
Paranoid mental disorders	8 (5.8)	9 (5.8)		
Bipolar disorder	3 (2.2)	8 (5.2)		
Mental disorders due to epilepsy	4 (2.9)	4 (2.6)		
Mental retardation with mental symptoms	14 (10.1)	9 (5.8)		
Other mental disorders	5 (3.6)	12 (7.8)		
Care assistant workers, n (%)			18.33	< 0.001
Primary health workers	26 (18.7)	34 (22.1)		
Community policemen	14 (10.1)	31 (20.1)		
Community cadres	89 (64.0)	63 (40.9)		
Volunteer and others	10 (7.2)	26 (16.9)		
Care willingness n (%)			2.24	0.69
Strongly willingness	29 (20.9)	31 (20.1)		
Willingness	69 (49.6)	75 (48.7)		
Neither willingness nor Unwillingness	31 (22.3)	38 (24.7)		
Unwillingness	5 (3.6)	8 (5.2)		
Strongly unwillingness	5 (3.6)	2 (1.3)		

Data were indicated by mean, standard deviation (SD), frequency and proportion

### Effect of training on perceived stigma and clinicians attitudes to mental illness

**Fig. 2:**
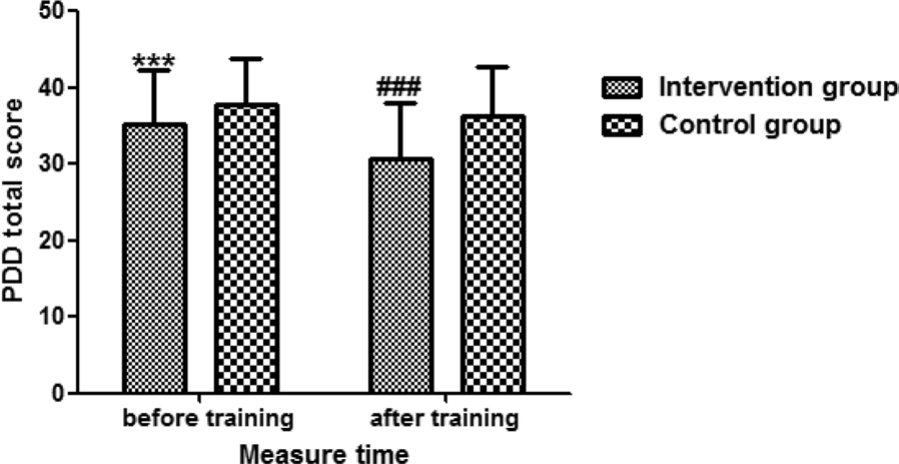
The effectiveness of anti-stigma training on PDD total score between the two groups. Education level, care assistant workers, and the baseline score of PPD were adjusted. *PDD* Perceived Devaluation and Discrimination Scale. Compared with the control group before training, ***P < 0.001. Compared with the control group after training, ^###^*P *< 0.001

**Fig. 3:**
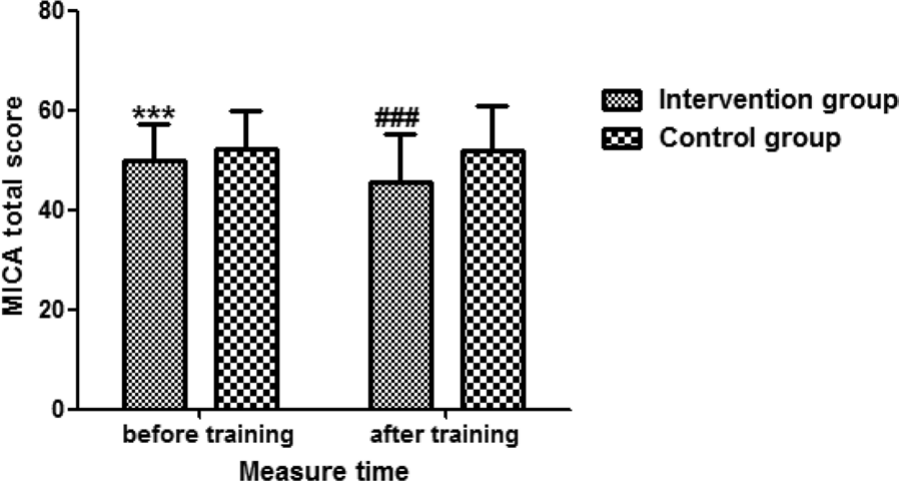
The effectiveness of anti-stigma training on MICA total score between the two groups. Education level, care assistant workers, and the baseline score of MICA were adjusted. *MICA* Mental illness: Clinicians’ Attitudes. Compared with the control group before training, ***P < 0.001. Compared with the control group after training, ^###^*P *< 0.001

As shown in Figs. 2, 3, the average score of PDD 35.3 (SD = 6.9) in the intervention group was significantly lower than the score 37.7 (SD = 6.0) in control group before training (*P *< 0.05). Similar to the MICA, the average score of MICA 49.9 (SD = 7.6) in intervention group was significantly lower than the score 52.4 (SD = 7.7) in control group before training (*P *< 0.05).

Multilinear regression models showed that participants in the intervention group had significantly lower scores of PDD 30.6 (SD = 7.4) after the training when compared with the control group 36.3 (SD = 6.4) (*Ab *= − 4.32, 95% *CI* from − 5.72 to − 2.93, *P *< 0.001). Additionally, there was a significant lower MICA score in intervention group 45.8 (SD = 9.5) after the training when compared with the control group 52.1 (SD = 8.8) (*Ab *= − 4.34, 95% *CI* from − 6.06 to − 2.62, *P *< 0.001) after adjusting for education level, care assistant workers, and the baseline scores of the PPD and MICA.

### Effect of training on stigma related knowledge

There were no significant differences in MAKS total scores in the intervention group 22.8 (SD = 2.6) when compared with the control group 23.1 (SD = 2.5) (*P *= 0.452). Multilinear regression models showed that there was no significant difference in stigma related knowledge as measured by MAKS total score between the intervention and control groups after training (*P *= 0.118) after adjusting for education level and care assistant workers.

**Fig. 4:**
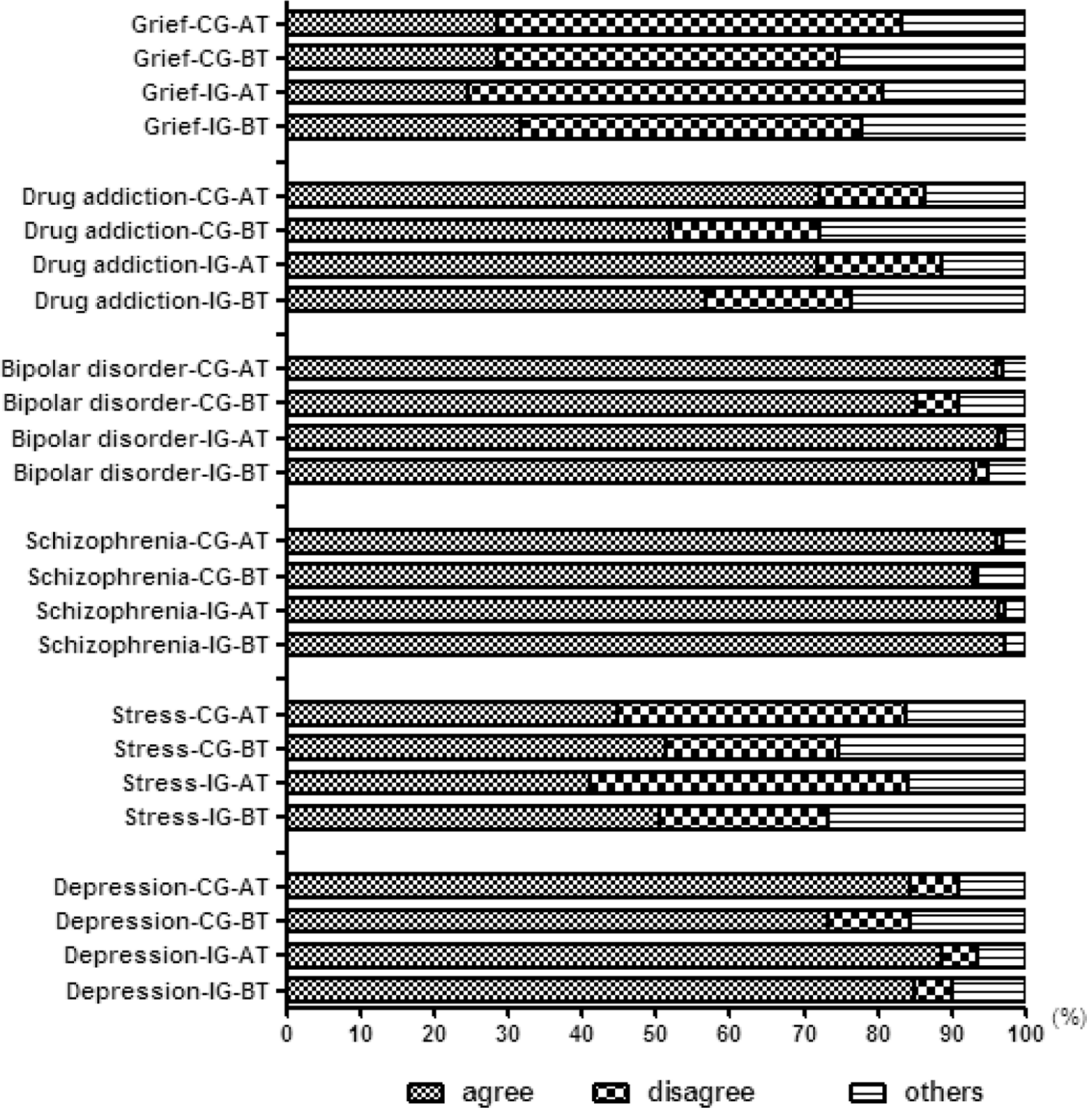
The percentage on identifying specific psychiatric disorders among care assistant workers. Agree: including strongly agree and slightly agree. Disagree: including slightly disagree and strongly disagree. Others: including neither agree nor disagree and don’t know. *AT* after training, *BT* before training, *CG* control group, *IG* intervention group

In order to measure the correct identification of psychiatric disorders, responses to items 7–12 of the MAKS were divided into agree (including strongly agree and slightly agree), disagree (including slightly disagree and strongly disagree) and others (including neither agree nor disagree and don’t know). As shown in Fig. 4, care assistant workers in intervention group had a high accurate rate of characterizing schizophrenia (> 95%), bipolar disorder (> 90%) and depression (> 85%) before and after training. Additionally, care assistant workers in the control group had a high accurate rate of characterizing schizophrenia (> 93%), bipolar disorder (> 85%) and depression (> 73%) before and after training. There was a significantly increase in those correctly identifying drug addiction as a psychiatric disorder among those in the intervention group after the training (from 57 to 72%, *P *= 0.009) and control group (from 52 to 72%, *P *< 0.001). The majority of care assistant workers (nearly 50%) in both groups did not correct identify stress and grief after training.

## Discussion

From more evidence-based policies to more evidence-based practices, provision of care for mental health problems has achieved significant development in recent years. The balanced care model increases service accessibility and task sharing relieves the human resource shortage in LMICs [2, 35]. However, care assistant workers are important members during task sharing, their service qualities could be influenced by the stigma and discrimination against people with mental illness [36]. To our best knowledge, this is the first study to conduct anti-stigma training among care assistant workers and explore its effectiveness on decreasing perceived discrimination, increasing mental health knowledge and improving attitudes towards mental illness in China.

From the perspective of Chinese collectivism culture, policies on mental disorders have significantly changed from the “public prevention and public treatment” in twentieth century to a social governance model—collaboration, participation and common interests—in twenty-first century. It points to the importance of protecting the human rights of people with mental disorders [37]. This is consistent with the goals of the Comprehensive Mental Health Action Plan 2013–2020 [38]. Care assistant workers could be a double-edged sword in delivering mental health care. The service quality could be influenced by their knowledge, attitudes and behaviours. Thus, interventions on care assistant workers are crucial. During this study, we found some encouraging improvements on the PDD and MICA scores after 3 h training, which showed that participants in the intervention group had significantly lower scores of PDD and MICA when compared with the control group after the training.

These results indicated that participants who received the anti-stigma training presented lower perceptions of devaluation-discrimination against people with mental illness and lower levels of negative stigma-related mental health attitudes. In our study, care assistant workers have good social contact with patients experiencing SMD by providing mental health care to them in their daily work. The education about anti-stigma may contribute to the positive outcomes to some degree. During the training, we explained the mechanism of stigma and discrimination and its negative consequences to the care assistant workers in order to renew their understanding of stigma and discrimination. The contents were formulated according to the effective interventions around the world and combined with the local mental health situations. Care assistant workers could understand and absorb the anti-stigma knowledge and skills easily. This might explain the positive changes after a short training. Our findings are consistent with those which have shown that attitudes can be improved following education aimed at reducing stigma [39, 40]. A systematic review of reducing mental health stigma among primary health care settings in LMICs demonstrated that education could have important impacts on stigma such as reducing stigmatizing attitudes [41]. Additional evidence indicates that education can positively change attitude, while there is less evidence for improving knowledge [42].

It is worth noting that care assistant workers in the study prioritized providing mental health care to individuals with SMD, such as schizophrenia, schizoaffective disorder, paranoid mental disorders, bipolar disorder, and epilepsy and mental retardation associated with mental disorders, which is consistent with the content of the “686 Program” [26]. And schizophrenia (nearly 70%) was the most common mental disorder in their management. Studies have shown that people with schizophrenia generally experience a higher level of stigma than other disorders [43, 44]. Public negative attitudes towards people with mental disorder are a potential source of self-stigma, which is produced in patients themselves [45]. Self-stigma and public stigma could produce a negative impact on the mental health help-seeking [46–48]. Care assistant workers share similar community culture and their positive attitudes could help patients increase their accessibility to mental health care. So, it is significant and meaningful to improve care assistant workers’ attitudes towards mental disorders in order to reduce the stigma among schizophrenia patients.

The pattern of the care assistant workers is a representative performance of multi-sectoral collaboration, which is necessary in promoting mental health care [49]. People with SMD are thought as dangerous, violent and unpredictable, which contribute to the close relationship between people with mental illness and the police [50]. Under the influence of deinstitutionalization and the development of the balanced mental health care model, more and more people are released from the hospital and enter community and this relationship seems to be strengthened [51]. In our study, care assistant workers are responsible for this group of patients who have potential violence to themselves, family or even society. From the perspective of public security, patients with SMD live in a mostly controlled environment. From the legal perspective, patients with SMD have the rights to get mental health care in a “least restricted environment” [52]. This is a very real social contradiction in China. Without keeping the balance between freedom and treatment, mental health care can have negative consequences. In our study, anti-stigma and discrimination training could help care assistant workers understand the principle of treating patients as the centre and treating patients with compassion and respect. Because of the small sample of the community policemen in this study, we did not analyze the specific effectiveness of the training on them. Further studies should explore the effectiveness among large number of the community policemen.

It is well known that lack of knowledge and negative stereotypes about mental illness could increase stigmatizing attitudes towards people with mental illness. In our study, we did not find any significant differences on MAKS total scores between the two groups. Both groups had a high score of MAKS at baseline, which means that they have a better understanding of the stigma-related mental health knowledge. This may be explained from the long-term health education in Guangzhou. Mental health education has been held in Guangzhou for more than 10 years, people could achieve the knowledge from print media and other multi-media. Abundant communication channels contribute to the widespread dissemination of mental health-related knowledge. There is a promise that stigma and discrimination towards people with mental illness could also be decreased through communication channels that spread anti-stigma knowledge and skills.

Additionally, the outcomes also showed that care assistant workers had better identification on schizophrenia, bipolar disorder and depression. And there was an increasing correct rate on recognizing drug addiction as a mental illness. However, nearly half of care assistant workers recognized stress and grief as mental illnesses in this study. These results indicate that some mental illness could be better understood after education, while there are still some complicated symptoms or emotional reactions which are difficult to understand and can easily be mistaken for mental illnesses. Without correct identification, individuals suffering from non-pathological mental health problems might be misdiagnosed as a mental illness, which may lead to stigmatization and discrimination [53]. There is a need to continue to improve mental health literacy among care assistant workers and help them distinguish mental health problems and mental illness.

### Strengths and limitations

Some limitations in this study should be considered. First, no follow-up assessments were conducted after the training. It is difficult for people to know whether the quality of mental health care improved after the training. Second, the intervention time to produce positive outcomes may be different according to different participant groups. The study also has some strengths. First, care assistant workers in this study are the new pattern of mental health care delivery in China. Second, this is the first study about anti-stigma training on care assistant workers in China, which may have some effects on community mental health care. Third, the contents of the training are developed according to the latest document and guidelines, which could be practical and repeatable.

## Conclusion

Training care assistant workers to deliver mental health care could bridge the shortage of human resources, which may result in positive treatment outcomes for people with mental disorders [11, 54]. Training care assistant workers with knowledge and skills to combat stigma could reduce their perceived discrimination and improve the attitudes towards people with mental disorders. Stigma and discrimination is the product of historical-cultural context [55]. Long term and multidimensional education on reduction stigma and discrimination is necessary in the future.

## Abbreviations

LMICs: low- and middle-income countries
SMD: severe mental disorders
PTSA: Policy, Training, Service, and Assessment
PDD: Perceived Devaluation and Discrimination Scale
MICA: Mental illness: Clinicians’ Attitudes
MAKS: Mental Health Knowledge Schedule
CI: confidence intervals
SD: standard deviation
